# Use of the Patient-Identified Top Health Priority in Care Decision-making for Older Adults With Multiple Chronic Conditions

**DOI:** 10.1001/jamanetworkopen.2021.31496

**Published:** 2021-10-28

**Authors:** Claire Davenport, Jennifer Ouellet, Mary E. Tinetti

**Affiliations:** 1 Brookdale Department of Geriatrics and Palliative Medicine, Icahn School of Medicine at Mount Sinai, New York, New York; 2 Department of Medicine, Yale School of Medicine, New Haven, Connecticut; 3 Department of Chronic Disease Epidemiology, Yale School of Public Health, New Haven, Connecticut

## Abstract

This cross-sectional study assesses how patient-identified top health priorities can be incorporated into the care of older adults with multiple chronic conditions while facilitating decision-making that aligns with these priorities.

## Introduction

Decision-making for older adults with multimorbidity is complicated by the uncertain benefits of many disease-based interventions.^1^ Variability in health outcome goals and health care preferences further confounds decision-making for this population.^1,2^ To address these issues, we developed Patient Priorities Care (PPC), which involves identifying and aligning care with patients’ specific health priorities.^1,2,3,4^ PPC has been associated with a reduced treatment burden and increased preference-concordant care.^1,5^ The goals of this cross-sectional study were (1) to determine the top health priority that patients most wanted to focus on to achieve their goals and (2) to consider how this priority addresses challenges while facilitating decision-making that aligns with patients’ priorities.

## Methods

The details of the PPC study, which was conducted from February 1, 2017 to August 31, 2018, have been described previously.^1,3,4^ This study comprised patients of 10 primary care clinicians in Connecticut. Patients were invited to participate if they were aged 65 years or older and had 3 or more chronic health conditions. An advanced practice registered nurse or case manager guided patients in identifying their health priorities, creating health priorities templates that included the following: (1) patients’ values; (2) up to 3 specific, actionable, and realistic health outcome goals; (3) up to 3 health care tasks that patients thought were doable and could help achieve their goals; and (4) up to 3 health care tasks that patients found burdensome or unhelpful. The Yale University Institutional Review Board approved this study. Oral informed consent was obtained from all participants. This study followed the Strengthening the Reporting of Observational Studies in Epidemiology (STROBE) reporting guideline.

The primary care clinicians were trained in aligning decisions with patients’ health priorities. After training, the clinicians and the PPC team participated in 21 case-based discussions.^3^ The group discussed how best to align care with patients’ health priorities, reported challenges, and suggested solutions to these challenges. One of the challenges identified was where to start aligning care. The proposed solution was to ask patients to identify the top health priority that they most wanted to focus on to achieve their goals.^1,3^ To create this patient-identified top health priority, patients were asked, “What 1 [health-related problem] do you most want to focus on so that you can achieve [health outcome goal] more easily or often?” Their responses were included in the template for clinicians to use in communication and decision-making. All patient responses are listed in the eAppendix in the Supplement.

One author (M.E.T.) reviewed patients’ responses, quantifying the health-related problems and suggesting how the patient-identified top health priority could address challenges and guide decision-making. Based on their review of the participants’ self-identified priorities and health outcome goals and informed by their experience in caring for older adults with multiple chronic conditions, the authors identified, discussed, and agreed on clinical challenges and decisional guidance. All authors reviewed and edited these documents iteratively until consensus was reached.

## Results

Of 236 eligible patients, 163 (69.1%) agreed to participate. This study included the 129 participants who were enrolled after the patient-identified top health priority was added. The mean (SD) patient age was 78 (7.6) years. Of the 129 participants, 125 (96.9%) self-reported as White and 86 (66.9%) were women, with a median of 4 chronic conditions (IQR, 3-5) and 7 prescription medications (IQR, 5-9).

Of the 129 participants, 127 (98.4%) identified the health-related problems on which they most wanted to focus (Table 1). Eighteen participants (14.0%) mentioned 2 problems. Eighty-two participants (64.6%) linked their health problem to actionable health outcome goals. Some patients identified specific interventions that they thought would address their top health priority and achieve their outcome goal (eg, “I want less pain in my knees so that I can walk more…. If I am not able to have the knee replacement, I’m not sure what is next”).

**Table 1.** Examples of Patient-Identified Top Health Priorities Reported by 129 Older Adults With Multiple Chronic Conditions^a^

Health problem reported^b^	No. (%)^c^	Example of patient-identified top health priorities reported
**Symptom or impairment**		
Musculoskeletal pain	42 (32.5)	I would like to have less pain so that I can do some renovations around my house. I know that I won’t get rid of the pain but less would be better.
		The pain in my hands. I make crafts and sell them to have spending money each month.
		My arthritis pain in my hands so that I can cook and cut up things easier. It is hard.
Fatigue	16 (12.4)	I want to be less tired so that I can walk more with my husband and have more energy in the mornings.
		I want to be less tired and loopy so that I can have energy to do household chores and cook again.
		I would love to get rid of my fatigue so that I can be able to go places and not feel fatigued.
Dizziness, imbalance	9 (7.0)	Check on my medications so that I can feel less dizzy when doing yard work.
		I want to know what is causing my equilibrium to be off so I can continue to be active and not fall.
		Be less dizzy and have less hip pain so that I can go out to breakfast and see my friends.
Mobility or walking impairment	7 (5.4)	I want to continue to be able to be mobile so that I can go to the dining room and talk with others.
		Improving my balance and my neuropathy so that I can walk outside of the building. I want to keep going shopping and to my grandchildren’s games.
		I would want to walk better by having less knee pain so that I can take care of my plants and garden.
Frequent urination or incontinence	6 (4.7)	I want to have less incontinence at night so that I can have more energy to go out and go to church and be awake to read.
		I would like to take less of the water pill so that I can get out more during the daytime to see friends and take trips.
Strength or range of motion impairment	5 (3.9)	I would like to increase the range of motion in my left shoulder so that I can hold my great grandson.
		… [M]y torn meniscus in my knee so that I can sleep better and walk longer.
		I wanted to improve the strength in my arms to lift things and play with my grandson.
Shortness of breath	4 (3.1)	I want to breathe better so that I can do things with less struggle, be active and go and do things. If I can’t, there is no sense in being here.
		I want to be able to breathe better so that I can keep going to the basketball games.
		I would like to breathe better so that I can mow my lawn, take care of my home, and get out and exercise.
Gastrointestinal^d^	5 (3.1)	I want to know if my medications are causing more fatigue and nausea so that I can do more housework and maintain my own home.
Anxiety, depression, mood	3 (2.3)	I want better control of my anxiety so that I can better control my blood pressure and maybe take less meds for blood pressure.
		I want to be less depressed so that I can travel more.
Visual impairment	2 (1.6)	I want my vision to improve so that I can do the things that I used to like cross-stitch and crossword puzzles… and keep volunteering.
**Health condition**		
Weight	11 (8.5)	Weight loss, and I want to have more stamina so that I can walk and work outside with less tiredness.
		I want to lose weight so that I can walk more.
		I used to walk my dog and see people in the complex, but I don’t have her anymore. Now it is hard with this weight.
Impaired sleep	9 (7.0)	I would like to sleep better so that I can have more energy and be awake in the mornings.
		I would like to be able to sleep a full night so that I can have more energy in the morning, during the daytime, and for travel.
Diabetes	7 (5.4)	My blood sugar scares me because it is hard for me to control. I want to have more energy. It has dropped down to 50 before.
		I would like to lower my diabetes medications so that I can avoid the problems that diabetes does to you, like dizziness, vision loss, and problems with feet and walking.
		I wish I didn’t have diabetes so that I can eat more candy at home and on vacation.
Neuropathy	5 (3.9)	I don’t want my neuropathy to get any worse. I can still feel my feet when I am driving.
		My neuropathy and my nerves so that I can have less pain when doing housework and activities.
Hypertension	3 (2.3)	I want better control of my anxiety so that I can better control my blood pressure and maybe take less meds for blood pressure.
		I would like to lose weight so that I can lower my blood pressure med and have more energy.
Other^e^	NA	NA
**Health care task**		
No. of medications	17 (13.2)	I would like to be taking less medication and seeing less specialists to have more time to go out with my husband and see my children.
		I would like to take less medications so that I can have less side effects like dry mouth and muscle pain.
		I would like to take less medications so that I can have less side effects, less to manage, maybe less constipation, and more time to go for a walk.
		I would like to trial less medication. If I have less medications, maybe I can have a normal day. My anxiety is raising my blood pressure because taking all these pills is too much.
Specific medication	6 (4.7)	I would like to take less of the water pill so that I can get out more during the daytime to see friends and take trips.
		I would like to get off the blood thinner so that I [do] not have to worry about bleeding [and] I can shave again. And it costs too much.
		I want to reduce metoprolol then taper off so that I can walk more briskly and up hills without my blood pressure dropping.
Glucose monitoring	2 (1.6)	Having to check my blood sugars 4 times a day because I have to worry about how much insulin; it depends on how much activity I do and what I eat.
		All of these needles so that I can go out to dinner without feeling like I have to bring a pharmacy.
Diet	2 (1.6)	I want to be able to be more disciplined with my meals so that I can be able to take trips with the senior center and not worry about being hungry.
		I want to go out to eat without so much worry about how the food is prepared and how that affects my diabetes.

Abbreviation: NA, not applicable.

^a^ The number of health problems reported totals to more than 129 because some participants reported more than 1 health problem. Of the 129 participants, 2 did not identify an actionable health problem they most wanted to focus on to achieve their goals (“I want peace of mind and financial security so that I can continue to volunteer and have a social life” and “I wouldn’t change a thing because I have such great doctors”).

^b^ Responses are to the following question posed by the member of the health team who guided participants in identifying their health priorities: “What symptom, health problem, or health care task do you most want to focus on to help you do [most desired goal activity] more easily or more often?”

^c^ Values are for the 129 participants who reported a health problem. All problems were included when participants reported more than 1. No participants mentioned more than 2 health-related problems.

^d^ Gastrointestinal problems included nausea, diarrhea, or abdominal discomfort.

^e^ Lymphedema, renal function, cancer, and fibromyalgia were each reported by 1 participant.

Table 2 displays the ways in which the patient-identified top health priority (1) addresses challenges in caring for persons with multiple chronic conditions and (2) facilitates patient priorities–aligned decisions.

**Table 2.** How the Patient-Identified Top Health Priority Addresses Challenges in Decision-making Among Older Adults With Multiple Chronic Conditions^a^

Challenge	How the patient-identified top priority addresses this challenge	How the patient-identified top priority exemplifies this challenge^b^	How the clinician can use the patient-identified top priority in communication and decision-making
**Uncertainty**			
Unclear what is most important in the face of multiple conditions	Focuses encounters and decision-making; identifies a place to start	“I want to know if my medications are causing more fatigue and nausea so that I can do more housework and maintain my own home.”	Start visits by addressing the priority: “I see that you are concerned about your medications causing fatigue. This is keeping you from your goal of maintaining your home. Let’s talk about what might be going on and what we can do to help.”
Uncertain applicability of disease guidelines	Concentrates shared decision-making around tradeoffs most important to the patient		
Uncertain benefits of interventions; patients vary in what matters most	Allows interpretation of guideline applicability through the lens of what matters most to the patient		Acknowledge uncertainty and the need for serial trials: “From reviewing your medications, I see several possibilities; it could be things other than medications as well. I suggest we check a few laboratory results and if they are okay, we can try switching to a different antidepressant as a first step. There are other things we can try later if needed.”^3^
	Addresses variability in what matters most		
**Conflicting perspectives**			
Across clinicians	Aligns everyone around addressing the health-related problem that matters most and achieving the patient’s health outcome goal(s)	“I want to reduce metoprolol then taper off so that I can walk more briskly and up hills without my blood pressure dropping.”	Align decision-making. Knowing what the patient most wants to focus on provides information for collaborative discussions and decision-making^3^: ^“^Given this patient’s goal to walk more briskly, can we discuss a trial of a lower dose or discontinuation of metoprolol, as it may be an important factor?”
Among family members or caregivers	Helps clinicians and families accept decisions that they may not otherwise agree with		
**Burdensome health care**			
Patient feelings of treatment-related burden	Supports removing unnecessary care or modifying necessary care to decrease burden	“I would like to be taking less medication and seeing less specialists to have more time to go out with my husband and see my children.”	Explore the reasons the patient feels burdened: “Can you tell me what it is about the number of medications and specialists you are seeing that is bothersome to you?”
Nonadherence as a result of burden	Improves adherence by linking recommendations to the patient’s goals		
Clinician discomfort with patients’ preferences not to accept recommendations	Helps clinicians accept the patient’s decisions		Acknowledge burden; link burdensome care to what matters to the patient: “I know you don’t want to continue seeing the pulmonologist, but it may be necessary to improve your breathing in order for you to achieve your goal of going out with your husband and seeing your children.”
	Can document informed choices		
**Inability to eliminate disease**			
Inability to eliminate symptom, impairment, or disease completely	Focusing on achieving the health outcome goal is often more successful than attempting to eliminate the symptom or impairment^6^	“This foot thing. I want to have more feeling in my feet so that I can drive safely and do more activities.”	Acknowledge that the symptom or impairment cannot be removed entirely but it may be possible to achieve health outcome goals: “We may not be able to completely resolve your foot pain and numbness, but the occupational therapist may be able to help with driving safety and other activities.”
	Achievement of health goal activity is a reliable metric of treatment success		Guide the patient to identify achievable goals based on the underlying value: “If a time comes that it won’t be safe for you to drive, we can discuss how you can continue getting around to visit your family and friends, which is most important to you.”

^a^ This information was derived through an iterative process aimed at identifying how the patient-identified top health priority address challenges in decision-making for older adults with multiple conditions while facilitating patient-aligned clinical decisions. The clinical challenges and decisional guidance were identified, discussed, and agreed on by the authors based on a review of patient-identified top health priorities and the health outcome goals of participants and were informed by experience caring for older adults with multiple chronic conditions.

^b^ All responses to the question “What 1 [health-related problem] do you most want to focus on so that you can achieve [health outcome goal] more easily or often?” are listed in the Supplement.

## Discussion

Like the traditional chief concern, symptoms and impairments were the most frequently mentioned health-related problems in this study. Linking these health-related problems to actionable outcome goals provides a platform for cross-condition decision-making for persons with multimorbidity.

The primary limitation of this study is that participants were drawn from a single site; therefore, studies of more diverse populations are needed. Work is ongoing to better understand how to align care with patients’ health priorities and to determine the effects of such alignment. Identifying the top health priority that patients want to focus on holds promise as an approach to initiating patient priorities–aligned decision-making, filtering all care through the lens of what matters most to each patient.

## Notes

Supplement. **eAppendix. **Patient-Identified Top Health Priority Responses
