# Supplementary material for: Use of the Patient-Identified Top Health Priority in Care Decision-making for Older Adults With Multiple Chronic Conditions
Source: JAMA Netw Open. 2021 Oct 28;4(10):e2131496. doi: 10.1001/jamanetworkopen.2021.31496 (PMC8554637; doi:10.1001/jamanetworkopen.2021.31496)
Supplement: Supplement. — eAppendix. Patient-Identified Top Health Priority Responses [file jamanetwopen-e2131496-s001.pdf]

# Supplementary Online Content

Davenport C, Ouellet J, Tinetti ME. Use of the patient-identified top health priority in care decision-making for older adults with multiple chronic conditions. *JAMA Netw Open*. 2021;4(10):e2131496. doi:10.1001/jamanetworkopen.2021.31496

Supplement. **Patient-Identified Top Health Priority Responses**

This supplementary material has been provided by the authors to give readers additional information about their work.

© 2021 Davenport C et al. *JAMA Network Open*.

Supplement. **Patient-Identified Top Health Priority Responses**

## All Responses for the ONE Thing among study participants

**Question posed**: What symptom, health problem, or healthcare task do you most want to focus on to help you do [most desired goal activity] more easily or more often?”

1. I would like someone to take a look at all of my meds to determine if they are working together to make me stronger or if some are a duplication so that I can go back to where I was two years ago, I was driving, walking with a cane and able to get out to the mall and to see my granddaughter's dance competitions.

2. Having to check my blood sugars four times a day because I have to worry about how much insulin I can give myself, it depends on how much activity I do and what I eat.

3. Pt states "The jumping pain in my legs", Spouse states "his legs jump and twitch during the night". Pt states, "my legs start to jump when I try to get up from the chair sometimes.

4. The pain in my back is getting unbearable, I had to shift to one side for two and a half years and it caused a curve in my spine and my shoulder to droop, putting pressure on my back.

5. My left hip and muscle pain so that I can get out more with my wife.

6. I would like less pain in my knees, especially when it rains.

7. He would like to discuss losing weight or having surgery to reduce his pannus (hx significant weight loss via bariatric surgery) in order to achieve his goals of being more active with his wife.

8. I get tired too easy but I am getting up a lot in the middle of the night.

9. "The dizziness that I am having in the morning.

10. Reducing this constant burning pain.

11. I would like to be taking less medication and seeing less specialists to have more time to go out with my husband and see my children.

12. My arm pain and pain in the back of my legs make it hard for me to get up and move around which I want to be able to do everyday.

13. My bladder,(Incontinence) I fell asleep on the chair last night and when I woke up it was a mess." I am getting dizzy, Oh, I have the vertigo, I was doing the leaves and things got fuzzy and I was losing my balance.

14. "I would like to stay the way I am [in terms of energy and back pain/arthritis] so that I can continue caring for my own home and Max."

15. I would love to see my kidney function improve more, I went from 19% to 25%, Dr. X was tickled that it improved.

16. Improvement in my lymph edema and less need for the wraps.

17. My hand mobility, I have trouble picking things up and my wife has to cut my food, my friends made me a card holder so I can still play with them.

18. Stop losing weight. I’m still losing weight, I don't know why, I am down to 143, it won't stop, my clothes are falling off, I eat spaghetti three times a day and drink the shakes, plus I have to worry about 'the feeling' and keep food with

© 2021 Davenport C et al. *JAMA Network Open*.

me all the time. I check my sugars 6 to 7 times per day out of worry, I went into a diabetic coma once and passed out on the heater. Dr. X said, he can't believe I survived.

19. I want to stay in remission, have less bone pain in my shoulder which I think in covered in legions, and be thinner.

20. Less muscle pain in my hands.

21. I would like to see less pain in my knees and my hands.

22. This dizziness that I have in the morning.

23. I want to have less pain in my back, even if I can get a little relief to do my own groceries. It's frustrating that I have to sit down every 10 minutes when I am trying to cook a meal for myself.

24. This diarrhea is terrible. Dr. X sent me to a GI to be sure but I am losing weight and have to wear pads.

25. I would like to have some lower back pain relief, it shoots down my legs and I can't walk to the end of my street anymore.

26. My bloating and digestion issues, I have to carry antacids with me at all times, I take turns, Pepcid, Pepto Bismol, and others to try and help.

27. I can't breathe enough to go swimming anymore.

28. My diarrhea is worse, I am now going four times a day since I started taking Miralax.

29. I want to have less incontinence at night so that I can have more energy to go out and go to church and be awake to read.

30. I still have pain in my hands, the right hand has improved, I can almost close it now, but it didn't help the left one.

31. The everyday pain in my side, it has gotten better over the years but it is still there.

32. My muscle pain and spasms, the pain travels up my leg and my legs jump when it happens.

33. This pain that I have on the left side of my lower back that travels down to the front of my leg, down to my knees, I can't go for a walk because of this pain.

34. would like to have less pain so that I can do some renovations around my house, I know that I won't get rid of the pain but less would be better.

35. I would like to lose weight so that I can maybe lower my blood pressure med and have more energy.

36. Managing my diabetes is hard, I don't want to go insulin.

37. I don't want my neuropathy to get any worse; I can still feel my feet when I am driving.

38. The pain in my back so that I can get around better, if I can get in and out of most cars I can go more places with friends"

39. I would like to have less lower back pain so that I can travel to New Hampshire twice per year to see my daughter.

40. I would like to increase the range of motion in my left shoulder so that I can hold my great grandson.

41. I would love to be able to come off these medications but they do help my essential tremors"

© 2021 Davenport C et al. *JAMA Network Open*.

42. Weight loss and I want to have more stamina so that I can walk and work outside with less tiredness.

43. I hope I have less pain and discomfort because of this compression fracture.

44. My neuropathy and my nerves so that I can have less pain when doing housework and activities.

45. I do have a lot of muscle pain but the medications are working.

46. increase my endurance so that I can do more around the house, I have major aches in my hips when I am standing long periods and walking.

47. I want to lose weight so that I can walk some more" "I used to walk my dog and see people in the complex but I don't have her anymore, now it is hard with this weight.

48. My arthritis in my hands so I can keep working and care for myself, I can't even make a fist anymore.

49. This foot thing, I want to have more feeling in my feet so that I can drive safely and do more activities.".

50. "have imbalance right now because of my vision it has been a rough go.

51. I would love to get rid of my fatigue so that I can be able to go places and not feel fatigued, take day trips" "fatigue for me is how hard it is getting up in the morning and to get moving.

52. This morning sickness, after I have breakfast, my eyesight gets blurry but I don't know why; sometimes my heart is beating fast then I may take a quarter, some days a half of a Metoprolol, sometimes once a day, sometimes twice.

53. All of these needles so that I can go out to dinner without feeling like I have to bring a pharmacy with me.

54. The aching in my body gets so severe at night that I am sensitive to touch, so that I can fall asleep sooner.

55. I want them to check if I have any medications that are causing side effects so that I can continue living a normal life on my own as long as possible,"

56. The pain in my hands, I make crafts and sell them to have spending money each month.

57. II pee through everything, I don't even feel it; it bothers me because it makes more laundry and work for my daughter.

58. I want to know if my depression medications or the combination of medications with my depressions meds are making me have cloudy thinking, word finding difficulty and some confusion"

59. Check on my medications so that I can feel less dizzy when doing yard work.

60. I wouldn't change a thing because I have such great doctors.

61. If my lower back pain were better I might try to golf again.

62. I want to know what is causing my equilibrium to be off so that I can continue to be active and not fall.

63. I want piece of mind and financial security so that I can continue to volunteer and have a social life.

64. I want to get off insulin, I don't think it is helping, my blood sugar is no different after I take it.

65. I would want to walk better by having less knee pain so that I can take of my plants and garden.

66. I want to have less pain in my knees so that I can walk around better with my great grandchildren.

© 2021 Davenport C et al. *JAMA Network Open*.

67. I would like to take less medications so that I can have less side effects like dry mouth and muscle pain.

68. I want to have to pee less so that I can: travel without stopping so much, as soon as I stand up I have to go.

69. I would like less knee pain so that I can: get around better and fall less from taking steps.

70. The urine, if they clear that up I will feel better mentally and physically it is very uncomfortable, but my feet are giving me a hard time, the pain and swelling in them makes it hard to go out

71. I want less pain in my knees so that I can walk more, I used to walk around the trailer park and at XXX Park. If I am not able to have the knee replacement, I'm not sure what is next.

72. I want to know if my medications are causing more fatigue and nausea so that I can: do more housework and maintain my own home.

73. I want better control of my anxiety so that I can better control my blood pressure and maybe take less meds for blood pressure.

74. would like to lose weight so that I can do more walking and have more energy.

75. I want to reduce my medications so that I can: alleviate worry about the side effects I'm having.

76. I would like to sleep better so that I can have more energy and be awake in the mornings. I don't fall asleep sometimes until 3 AM or 4 AM and can't get up in the mornings.

77. I would like to get off the blood thinner so that I can: not have to worry about bleeding, I can shave again and it costs too much.

78. My blood sugar scares me because it is hard for me to control, I want to have more energy, it has dropped down to 50 before

79. I would like more regularity with by bowel and bladder, sometimes I'm hard, sometimes I'm soft.

80. worry less and sleep better it does interrupt my sleep.

81. I want less pain and weakness in my right knee so that I can: walk more, maybe without the cane and travel in more comfort.

82. My arthritis pain in my hands so that I can: cook and cut up things easier, it is hard.

83. I want to reduce Metoprolol then taper off so that I can: walk more briskly and up hills without my blood pressure dropping.

84. I want some improvement with my [neuropathy so that I can be a bit more mobile. Can I ever recover from this, 'no' but I certainly would like to be a little bit more mobile?

85. I want to breathe better so that I can: do things with less struggle, be active and go and do things, if I can't there is no sense in being here.

86. I would like better balance.

87. I want to have less arthritis pain so that I can: have less discomfort during storms and knit more.

88. I would like better sleep so that I can: have energy to leave my apartment and go to the dining room. "If you want a good day you have to sleep.

© 2021 Davenport C et al. *JAMA Network Open*.

89. I want help managing my new diagnosis of Fibromyalgia and Restless Sleep so that I can: have less side effects of medications and keep doing my crafts.

90. I would like less back pain so that I can: live as independently as possible in my home and walk better.

91. I want to lose weight so that I can: not get diabetes and have more energy.

92. My leg cramps and my torn meniscus in my knee so that I can: sleep better and walk longer.

93. Be less dizzy and have less hip pain in the morning so that I can: go out to breakfast and see my friends. When you are tired and dizzy, you don't want to go out. The hip doesn't hurt when I am sitting, only when I move.

94. I want less back pain and dizziness so that I can: keep living at home and do more with my husband around the house.

95. I want to take less medications so that I can: worry less about the side effects and keep taking care of myself.

96. I want less lower back pain so that I can: bend over with freedom and without pain so that I can pick things up off floor- I definitely need to address the weight problem, it is definitely influencing the lower back pain.

97. I want my right leg better so that I can: walk more and use the walker less.

98. I would like to lower my diabetes medications so that I can avoid the problems that diabetes does to you, like dizziness, vision loss and problems with feet and walking.

99. I want to have more energy in the mornings so that I can continue to walk my dog, work and get my granddaughter off the bus.

100. I want to be less tired so that I can: walk more with my husband and have more energy in the mornings.

101. I want less leg and knee pain so that I can see my new grandbaby without pain and have less when I am painting.

102. I want to feel less groggy in the morning and have less knee pain so that I can: do the activities that I set out to do each day and be active.

103. I would like to get this ankle healed so that I can walk without excruciating pain when I am helping my daughter and granddaughter.

104. I would like to be able to sleep a full night so that I can: have more energy in the morning, during the daytime and for travel.

105. I want my vision to improve so that I can: do the things that I used to like counted cross stitch and cross word puzzles, I also use social media to connect with family that lives out of state and keep volunteering.

106. I want to continue to cut back on my medications so that I can have less numbness and tingling and less side effects to medications.

107. I want to continue to be able to be mobile so that I can: go to the dining room and talk with others.

108. I want to be able to be more disciplined with my meals so that I can be able to take trips with the senior center and not worry about being hungry. I am eating plenty but my stomach cannot take more than two hours.

109. trial less medication so that I can: If I have less medications maybe I can have a normal day. My anxiety is raising my blood pressure because taking all these pills is too much.

© 2021 Davenport C et al. *JAMA Network Open*.

110. I would like to go out to eat without so much worry about how the food is prepared and how that affects my diabetes.

111. Improving my balance and my neuropathy so that I can: walk outside of the building, right now I can only walk inside. I want to keep going shopping and to my grandchildren's games.

112. I want to be able to breathe better so that I can keep going to the basketball games, I don't want anything to get in the way of that.

113. I wish I didn't have diabetes so that I can: eat more candy at home and on vacation.

114. I want to be able to sleep and not be fatigued when I wake up so that I can have more energy and plan to do things with my husband.

115. I would like to try and take less medications so that I can: have less side effects, less to manage, maybe less constipation and more time to go for a walk.

116. I want to lose weight so that I can walk more outside with my neighbors and have a better quality of life.

117. I want to be less depressed so that I can travel more. I went to Orlando and Trinidad last year. I want to travel to Hollywood to see my granddaughter dance.

118. I want less arthritis pain and diabetes to go away so that I can be able to take care of my granddaughter.

119. I would like to lose weight, and have my Neuropathy more consistently controlled so that I can have more energy, feel a little bit better about myself and do activities with less discomfort.

120. I want to lose weight so that I can accomplish all the things on my list, help my blood pressure, have more energy to visit my daughter away at school and do more in a shorter amount of time.

121. I don't want to take a lot of medications because I don't want to lose control and not be able to get out to the senior center and dance.

122. I would like to lose weight and have less knee and shoulder pain so that I can be active with my grandson and travel.

123. I want to get off some of these medications that are making me feel tired so that I can go out shopping with my niece and nephew.

124. I would like to breathe better so that I can mow my lawn, take care of my home and get out and exercise.

125. I want less lower back pain so that I can have less pain while gardening and lifting when I am with community members.

126. I would like to take less of the water pill so that I can get out more during the daytime to see friends and take trips. It makes me go too much during the daytime. I have to wait to take it until after I go to church and until after I go to the hairdresser.

127. I wanted to improve the strength in my arms to lift things and play with my grandson.

128. I would love to lower my statin so that I can less muscle pain in the back of my legs and when I am with my grandchildren and golfing.

129. I want to be less tired and loopy so that I can have energy to do household chores and cook again.

© 2021 Davenport C et al. *JAMA Network Open*.
